# Classification of Post‐Delousing Mortality in Farmed Atlantic Salmon: A Case Study of Standardised Causal Classification at Fish‐Level

**DOI:** 10.1111/jfd.14087

**Published:** 2025-01-31

**Authors:** Nanna K. Ringstad, Marit Stormoen, Paul J. Midtlyng, David Persson

**Affiliations:** ^1^ Department of Production Animal Clinical Sciences, Faculty of Veterinary Medicine Norwegian University of Life Sciences Ås Norway

**Keywords:** cause‐specific mortality, injuries (trauma), macroscopic pathology, mechanical delousing, salmon farming

## Abstract

The study investigated cause‐specific, fish‐level mortality of farmed Atlantic salmon following mechanical delousing. We visited three populations at two marine sites belonging to one company at four different time points, from 1 day before to 13 days after the mechanical delousing. A total of 453 dead fish were collected and necropsied during the four visits. The underlying mortality cause was determined by macroscopic pathology in fish that died peracutely, acutely and subacutely after the procedure. The macroscopic pathological changes were recorded, and a cause of death was assigned to each fish by applying a uniform code system to classify mortality and losses in farmed Atlantic salmon. The findings revealed that mechanical delousing procedures can lead to mortality and pathological changes up to 13 days after the procedure. We identified cerebral haemorrhages in approximately 25% of the necropsied fish that died peracutely. Comparing the cause‐specific mortality assignments at necropsy to daily mortality recordings by farmers showed that farmers assigned a smaller proportion of the fish to injury/trauma than the necropsy indicated. This implies that current practices for recording mortality causes by farmers after delousing may overlook the long‐term negative effects of the procedure. Therefore, guidelines for farmers to more accurately record mortality causes following delousing are necessary to improve the value and precision of these records.

## 1 Introduction

In 2023, the mortality incidence among Atlantic salmon reared in Norwegian marine aquaculture was 16.7%, according to the Norwegian Fish Health Report: this corresponds to 62.8 million salmon out of approximately 300 million seawater‐reared individuals (Sommerset et al. 2024). The high mortality rate raises animal health and welfare concerns and negatively affects the industry's cost‐effectiveness and sustainability. The annual Norwegian Fish Health Report mainly attributed these mortalities to injuries from delousing procedures and infectious diseases such as gill disease, winter wounds ( *Moritella viscosa* ) and Cardiomyopathy Syndrome (CMS) (Sommerset et al. 2024).

Recording daily mortality is mandatory for all Norwegian fish farms (Norwegian Ministry of Trade, 2008). In seawater farms, dead fish are retrieved from the bottom of the net pen and manually counted daily. Today, most farmers assign a cause to these mortalities in their production database. Health visits from fish health personnel (FHP) are typically performed monthly and are legally required in all Norwegian fish farms (Norwegian Ministry of Trade, 2008). The FHP examine a few dead or moribund fish from each pen during their visits and make recommendations to the farmers on the mortality causes and proportions that should be recorded in the coming month. The recommendations are based on clinical signs, necropsy findings, diagnostic results and recent events at the site, such as bad weather, handling or delousing operations (Vatne et al. 2024). Fish are rarely necropsied between the monthly visits by FHP.

In 2021, a standardised code list for mortality causes and losses in aquaculture was proposed. The list was developed by the Norwegian University of Life Sciences (NMBU) and commissioned by NCE Seafood Innovation. This standardised code list for salmon is developed to include causes for mortality and losses during production, but also for quality downgrading at harvest and causes for reduced growth (Aunsmo et al. 2023). Before its implementation, each company used their own lists of mortality causes, often lacking a unified structure and combining a mixture of recording clinical signs, risk factors and actual causes (Aunsmo et al. 2023). Inconsistent cause‐specific mortality registers caused a lack of clarity, efficiency and communication regarding the main health issues in aquaculture. Through an industry initiative, the cause‐specific mortalities are reported to a joint database (AquaCloud) that now covers more than half the Norwegian biomass of farmed salmon (Blom 2023). Daily mortalities are reported and classified in the database according to the standardised code list. For the first time, data from these industrial recordings were included in the annual Norwegian Fish Health Report published by the Norwegian Veterinary Institute for 2023. The main mortality causes in marine farmed salmon in 2023 recorded in the AquaCloud database were infectious diseases (38.1%), injuries (32.9%) and unknown causes (19.6%) (Sommerset et al. 2024).

The standardised list of mortality causes in salmon follows principles from the eleventh revision of the International Classification of Diseases (ICD‐11) used in human mortality registers (Harrison et al. 2021; World Health Organization 2019/2021). The ICD‐11 is strictly based on causality, where the underlying cause is the recorded cause of death. The underlying cause of death is defined by World Health Organisation (WHO) as ‘the disease or injury which initiated the train of morbid events leading directly to death, or the circumstances of the accident or violence which produced the fatal injury’ (WHO n.d.). Recording the underlying cause of death provides consistent and comparable information on populational health across time and geographical areas. A significant difference between human and salmon mortality registers is the focus on individuals in human registers. In human populations lacking medical death certification, verbal autopsies are used to estimate the distribution of mortality causes (World Health Organization 2007). Through standardised interviews in verbal autopsies, information about the deceased's symptoms, medical history and death circumstances is collected from next of kin or caregivers. Healthcare professionals or algorithms then analyse this data to determine the most probable cause of death (Chandramohan et al. 2021).

Mortality registers, in humans and animals, are valuable tools for disease monitoring, effective resource allocation and targeted health interventions. Therefore, ensuring good data quality in these registers is essential to make well‐informed decisions for population health. For instance, delousing and handling procedures aimed at removing salmon lice (*Lepeoptheirus salmonis*) are frequently named significant contributors to mortality in Norwegian salmon farming (Oliveira et al. 2021; Persson et al. 2021). A study by Sviland Walde et al. reported an increase in mortality following all types of delousing methods and found variations in mortality patterns for the different delousing methods (Sviland Walde et al. 2021).

Non‐medicinal methods (NMM) have become the most common delousing procedures in Norwegian salmon farming due to resistance development against chemotherapeutants (Myhre Jensen et al. 2020; Overton et al. 2018). Mechanical delousing methods, which use brushes and/or water jets to remove salmon lice, have become increasingly common, along with warm water (thermal treatment), long‐term freshwater baths and combinations of different methods. All these procedures involve handling the fish (crowding and pumping) to get the fish treated with NMM. This handling and the delousing procedure can lead to pathological changes such as skin injuries, gill bleeding, cerebral haemorrhage, scale loss and damage to eyes and fins (Bui et al. 2023; Thompson et al. 2023; Østevik et al. 2022).

Mechanical delousing was the most common delousing method used in Norway in 2023 (Sommerset et al. 2024), yet there is a large variation in how the ensuing mortalities are classified. Experience from the field indicates that both causal categories and the duration of the recordings associated with delousing can vary. Typically, companies attribute all mortalities between 1 and 5 days to the procedure. These variations complicate the comparison of mortality rates across sites, companies, methods and time.

This study aimed to identify the underlying cause of death in fish that died peracutely, acutely, and subacutely after a mechanical delousing procedure. Further, we wanted to evaluate how the standard for classifying mortality is best utilised with respect to the causal category and the duration of these recordings, following a mechanical delousing.

## 2 Material and Methods

### 2.1 Fish Populations

The selected two marine farming sites are owned by the same company and located in the archipelago west of Trondheim, Norway, in an aquaculture dense area. The sites were selected based on willingness to participate and accessibility for sampling, with the only inclusion criterion being a planned procedure of mechanical delousing. Three net pen populations of diploid Atlantic salmon situated in these two sites were sampled before and after a mechanical delousing procedure against salmon lice in the fall of 2023. Two groups (Populations 1 and 2) were located at the same site but were sampled 2 weeks apart.

Information on the health status of the fish populations was provided by the FHP before the study. In Populations 1 and 2, circulatory disease caused by piscine myocarditis virus (PMCV) or piscine orthoreovirus (PRV) was suspected but negative on PCR tests. According to the local FHP, mortality due to circulatory disease was low in both populations. In population 2, one fish tested positive for bacterial kidney disease (BKD) on PCR 2 weeks before the study, but the fish group did not show mortality or clinical signs of BKD. In Population 3, PRV had been detected at the site 4 months before the sampling commenced and mortalities attributed to heart and skeletal muscle inflammation (HSMI) had remained low since the virus was detected. This site also had a recurring issue with cachectic individuals (‘stunts’), deemed related to infectious pancreatic necrosis (IPN) virus infection during the hatchery phase. Despite frequent attempts to remove such fish earlier, a continuous low frequency of mortalities due to this condition occurred throughout the fall.

Because mortalities were low in all populations before the study, the FHP evaluated the risk associated with handling and delousing being low. Information on the population demographics and rearing history was obtained retrospectively from the production management system and is summarised in Table 1.

**TABLE 1:** Characteristics of the three fish populations included in the study.

	Population 1	Population 2	Population 3
Site	Site A	Site A	Site B
Delousing procedure	Hydrolicer	Hydrolicer	SkaMik
Fish count (Day 1 before delousing)	171,199	178,836	173,021
Fish count (Day 13 after delousing)^b^	170,641	177,635	172,466
Date of sea transfer	January 24, 2023	December 30, 2022	May 19, 2023
Days at sea^a^	281 days	324 days	182 days
Mean weight^a^	2.5 kg	2.9 kg	1.1 kg
Sea temperature^a^	9.8°C	8.6°C	9.10°C
Daily mortality (%) 13 days prior to study	< 0.01%	< 0.01%	< 0.02%
No. of previous delousing events	4	3	4
Health status	Circulatory disease suspected	Circulatory disease suspected	HSMI (piscine orthoreovirus) diagnosed

^a^ Parameters marked indicate values were recorded 1 day prior to the delousing.

^b^ Indicate that this is count after mortality.

### 2.2 Delousing Procedures

The fish in Populations 1 and 2 were deloused using Hydrolicer (SMIR n.d), while Population 3 was treated using SkaMik 1.5 (SkaMik n.d). Both methods are mechanical delousing technologies. The Hydrolicer removes the salmon lice from fish using low‐pressure water jets (Erikson et al. 2019), whereas the SkaMik system combines water‐flushing and brushing the lice of the fish (Westgård et al. 2021). All three populations were starved between 48 and 72 h before the delousing, corresponding to normal industry practices.

### 2.3 Sampling

Each fish population was investigated four times, from the day before treatment to 13 days after the delousing procedure. The visits were scheduled as follows: 1 day before the delousing (Visit 0), during the first retrieval of dead fish after the delousing (1 day after) (Visit I), 3 or 4 days after the delousing (Visit II) and 11 or 13 days after the delousing (Visit III). These days were chosen to represent the baseline (Visit 0), peracute (Visit I), acute (Visit II) and subacute (Visit III) mortality related to the delousing.

On certain days (for Populations 1 and 2), the site could not pump dead fish for one or more consecutive days due to weather conditions. Hence, the dead fish examined could have originated from 2 or 3 days earlier (see Table 2 legend). During the sampling visits, we attempted to examine all available dead fish and assign a cause of death to each individual. To exclude fish that had been dead for more than 24 h, the status and colour of gills were macroscopically evaluated on each fish. Moribund or euthanised fish were not included, except for visit I in Population 3, where seven moribund fish were euthanised (blow to the head) and mixed with the dead fish due to miscommunication during sampling. During some of the visits, the number of fish exceeded what could be examined in the available time frame. This occurred in Visit I in Population 1 and Visits I and III in Population 2. In these visits, fish were haphazardly chosen based on what was conveniently provided by the farmers and necropsied in order from top to bottom of the bunk. Table 2 presents the number of necropsied fish in the study and the total number of dead fish recorded in the production database for the days of the visits.

**TABLE 2:** Number of fish necropsied during the four visits and the number of fish recorded as dead in the production management system on the same days.

	Necropsy						Total mortality (1 day before to 13 days after)	
	Visit 0	Visit I	Visit II	Visit III	Total necropsied	Total mort. during visits	Dead fish	% acc. mortality
Pop. 1	3/5	126/360^a^	9/18^c^	17/26	155	409	558	0.33%
Pop. 2	0/2	92/667^a^, ^b^	13/19	52/175^a^, ^b^	157	863	1201	0.67%
Pop. 3	12/14	85/127	5/9	39/39	141	189	535	0.31%

*Note:* The total mortality in the study period after delousing in count and accumulated % mortality per fish group. Dead fish does not include euthanised fish.

^a^ Visits with more dead fish than what was achievable to examine during the visit.

^b^ Accumulations of mortality over 2 days.

^c^ Accumulations of mortality over 3 days.

The daily mortality percentage and cause‐specific fractions recorded by the farmers were collected retrospectively for the study period. This was also gathered for the 13 days before the delousing to compare mortality trends between the populations. These data relied on the accuracy of the farmer's routine mortality recordings.

### 2.4 Pathology and Mortality Cause Assignment

Each necropsied fish was subjected to a macroscopic pathological examination in the field. A set of predefined common external and internal pathological changes were recorded. External changes included visible defects (wounds, injuries or deformities), fresh wounds and petechiae on gills, and were recorded as present/absent. Shortened operculum and loss of scales were assigned a grade from 0 to 3 according to the proposed welfare standard in Laksvel 2022 (Nilsson et al. 2022). Internal changes were recorded as present/absent and included blood in the pericardium, ascites, discoloured liver, fibrin on the liver, petechiae on the liver, petechiae on internal organs, petechiae or haemorrhage in muscle and cerebral haemorrhage. Recent feed intake was checked for by examining the intestine. Cerebral haemorrhage only included visible haemorrhages in the cerebral tissue or the cranial cavity, not cerebral hyperaemia. Discoloured liver included pale, marbled and deviating colouration of the liver parenchyma. Sampling of tissue was not performed as part of the study.

**FIGURE 1:**
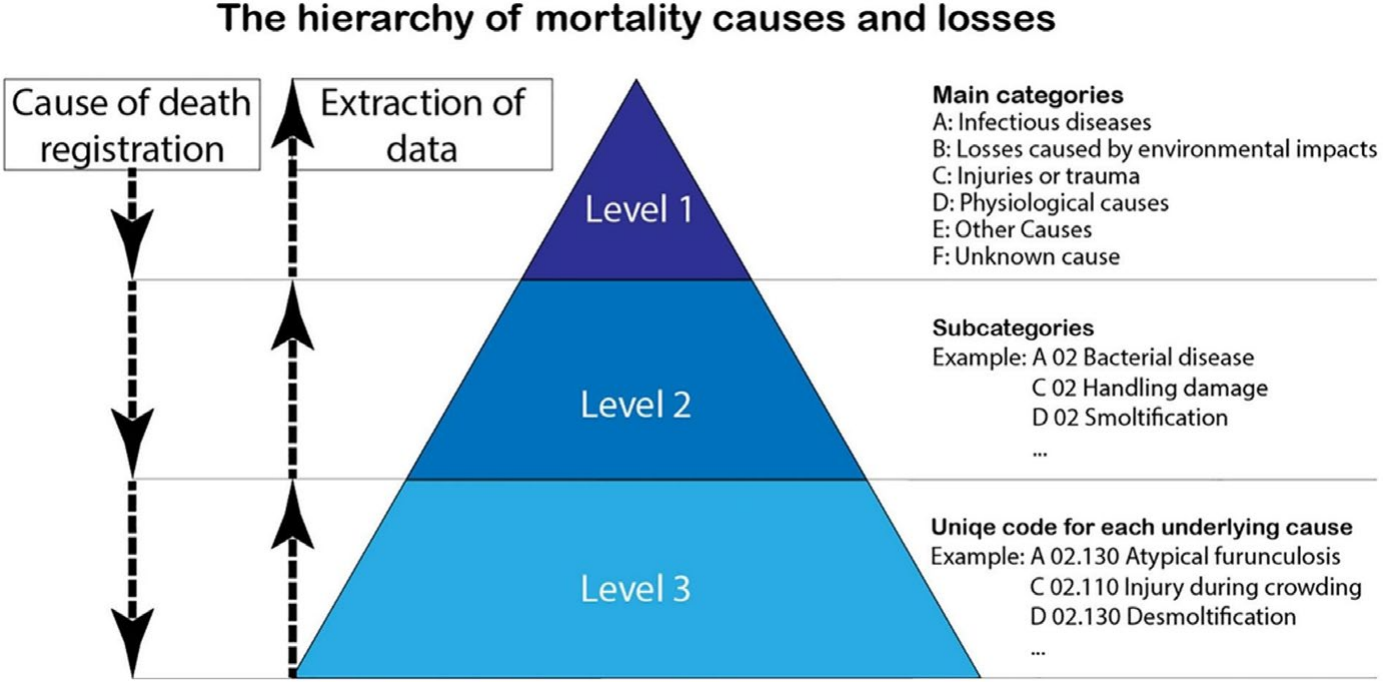
The standard for classification of losses and mortality causes in aquaculture has a hierarchical structure, with three levels for recording the cause of death. Reprinted from ‘Real‐time monitoring of cause‐specific mortality and losses in industrial salmon farming’ by Aunsmo et al. 2023, Aquaculture, 574, 737–748. © 2022 Aunsmo et al. Published by Elsevier B.V. Licensed under CC‐BY.

Based on necropsy findings, in addition to the clinical history of the populations, a cause of death was assigned according to the standard for classification of losses and mortality causes in aquaculture (Aunsmo et al. 2023). Briefly, the mortality classification system is organised hierarchically into three levels. At Level 1, there are six causal categories: (A) infectious diseases, (B) environmental impacts, (C) injury/trauma, (D) physiological causes, (E) other causes and (F) unknown causes. Level 2 includes subcategories based on causality and Level 3 contains an unlimited number of specific underlying causes (Aunsmo et al. 2023). The cause of death can be recorded at any of these levels (see Figure 1).

Based on the methods used in Aunsmo et al.'s study in 2008 (Aunsmo et al. 2008), the cause of death for each fish was assigned a likelihood grade. The likelihood was assigned on a scale from 1 to 3: (1) very likely, (2) likely and (3) the most likely among the differential diagnoses. The necropsy assignment of mortality cause and likelihood grading were performed by the same person for all necropsied fish to minimise inter‐observer variability.

### 2.5 Data Management and Analyses

The daily mortality (%) recorded by farmers was summarised over the days following the delousing procedure. Initial data collection was done in Microsoft Excel (Microsoft Corporation, Redmond, USA). All data analyses and cleaning were done in Stata18 (StataCorp, College Station, Texas).

## 3 Results

### 3.1 Necropsy Findings

During the four visits, 453 fish were examined and recorded for macroscopic pathology, and the most likely cause of death was assigned. Three causal categories (Level 1) were recorded in the necropsied fish: infectious diseases (category A), injury/trauma (category C) and unknown (category F). Table 3 presents the number of fish assigned to each of these categories (A, C and F) for each visit. Overall, the likelihood grade very likely (Grade 1) was attributed to 42% of the causes, likely (Grade 2) to 30% and the most likely among the differential diagnoses (Grade 3) to the remaining 28%.

**TABLE 3:** Number of fish from each population (Pop. 1–3) recorded in the three causal categories (A—infectious, C—injury and F—unknown). The table is organised by visit (0, I, II and III), with percentages within each visit and population. The number of fish per visit for each population is presented (Total), and the total number and percentage for the three causal categories is presented (Tot.). Pie charts represent the shares of the categories within each visit for the populations combined.

	Visit 0			Visit I			Visit II			Visit III			Tot.
	Pop.1	Pop.2	Pop.3	Pop.1	Pop.2	Pop.3	Pop.1	Pop.2	Pop.3	Pop.1	Pop.2	Pop.3	
Injury /trauma (Cat. C)	2 (66%)	0	4 (33%)	126 (100%)	84 (91%)	61 (72%)	8 (89%)	13 (100%)	3 (60%)	9 (53%)	24 (46%)	23 (59%)	*357 (79%)*
Infectious disease (Cat. A)	1 (33%)	0	0	0	8 (9%)	22 (26%)	0	0	0	2 (12%)	12 (23%)	9 (23%)	*54 (12%)*
Unknown (Cat. F)	0	0	8 (66%)	0	0	2 (2%)	1 (11%)	0	2 (40%)	6 (35%)	16 (31%)	7 (18%)	*42 (9%)*
Total	3	0	12	126	92	85	9	13	5	17	52	39	453
	15			303			27			108			
	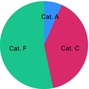			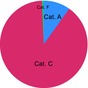			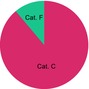			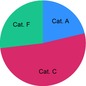			

Macroscopic pathology was observed in 82% (372 fish) of the necropsied fish. Each fish could have multiple pathological findings. Approximately 40% of the necropsied fish had external visible defects like wounds, injuries and deformities. Wounds were recorded in 61% of the fish with visible defects and had an overall occurrence of 24%. The most common macroscopic findings were discoloured liver (46%), cerebral haemorrhage (23%), blood in the pericardium (20%) and petechiae on gills (15%). All other macroscopic findings had an occurrence lower than 10%. Only four necropsied fish had feed in the intestine indicating that the vast majority had not regained appetite since the treatment event. During Visit I, approximately 60% of the necropsied fish had zero or one pathological change, while 12% had three or more. By Visit III, this shifted towards multiple pathological findings per fish, with 35% having zero or one pathological change and 40% having three or more.

#### 3.1.1 Visit 0 (1 Day Before Delousing)

Before delousing, mortality patterns had remained stable, with daily mortality (%) lower than 0.02% (mean 9.7 dead fish per day) in all three populations. A total of 15 fish were necropsied during Visit 0, accounting for 71% of retrieved dead fish. The causal category of mortality in these fish was primarily unknown and injury/trauma. Almost 90% of the causes in Visit 0 received the most certain likelihood grading, very likely (grade 1).

External visible defects were observed in 67% (10 fish) of the necropsied fish, and 70% (7 fish) of these fish had wounds. High grades of scale loss (Grade 2 or 3) were observed in 53% (8 fish) necropsied during Visit 0.

#### 3.1.2 Visit I (1 Day After Delousing)

In total, 303 fish were necropsied during Visit I, representing 26% of the total mortalities retrieved during this visit. Injury/trauma was the most recorded causal category in the necropsied fish, with 72%–100% of the fish assigned this category (see Table 3). The most common causes recorded during Visit I was the delousing method (Hydrolicer and SkaMik—51% of the assignments) and handling injury unspecified (35% of the assignments). In Visit I, there was an increase in the occurrence (%) of infectious diseases in Populations 2 and 3; see Table 3. These fish were recorded with HSMI or viral disease unspecified as mortality cause. The likelihood grades were evenly distributed between very likely (Grade 1), likely (Grade 2) and most likely among the differential diagnoses (Grade 3).

Macroscopic pathological changes observed during visit I included traumatic injuries to the skin, cerebral haemorrhage and petechiae on gills. Approximately 20% (65 fish) had petechiae on gills during Visit I, but this occurrence was not seen during the other visits (< 8%). External visible defects were observed in 23% (70 fish) of the necropsies, and 47% (33 fish) of these fish had wounds. During this visit, 65% (196 fish) were scored with a high degree of scale loss (Grade 2 or 3).

**FIGURE 2:**
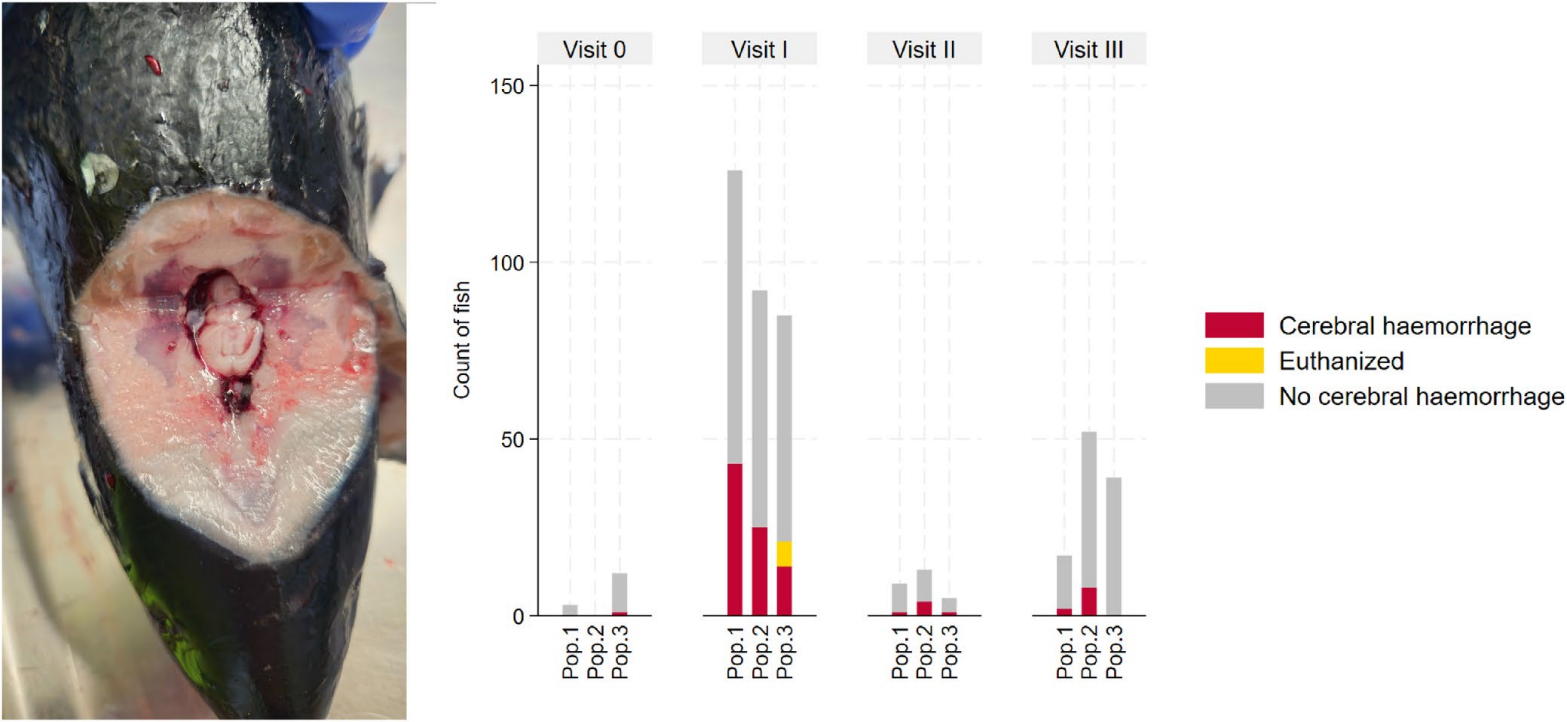
Left: Photo of an Atlantic salmon with a cerebral haemorrhage viewed from a dorsorostral angle. The skull roof is removed, showing the cranial cavity filled with blood surrounding the cerebral tissue. Right: Stacked bar graph showing the count of necropsied fish with (red) and without (grey) cerebral haemorrhage for each visit in the three fish populations (1–3). Fish from Population 3 that were euthanised by a blow to the head are marked as ‘euthanised’ (yellow). Figure created with STATA 18 and BioRender.com.

Cerebral haemorrhage was observed in all three populations, see Figure 2. At this visit, 34% (43 fish) from Population 1 and 27% (25 fish) from Population 2 had cerebral haemorrhage. In Population 3, 25% (21 fish) were recorded with cerebral haemorrhage during Visit I. However, as 7 fish were euthanised with a swift blow to the head, this number overstates the prevalence among the dead fish. Adjusting for this, the occurrence is reduced to 18% (14 fish) in Population 3.

#### 3.1.3 Visit II (3/4 Days After Delousing)

By Visit II, the daily mortality had stabilised at < 0.02% in all populations, the appetite had normalised and the groups had resumed feeding. In this visit, 27 fish were necropsied, which represented 59% of the retrieved fish. The high frequency of injury/trauma persisted in Visit II, where 60%–100% of the fish were recorded in this causal category (see Table 3). At Visit II, the mortality causes were recorded equally between very likely (Grade 1) and most likely among the differential diagnoses (Grade 3).

External visible defects were seen in 85% (23 fish) of the fish, and 60% (14 fish) of these were recorded with wounds. A high degree of scale loss (Grade 2 or 3) occurred in 52% of the fish (14 fish). Cerebral haemorrhage was also observed in all populations during this visit, but the occurrence was considerably reduced compared to Visit I (see Figure 2).

#### 3.1.4 Visit III (11/13 Days After Delousing)

At Visit III, mortality had slightly increased again in all groups. A total of 108 fish were necropsied, accounting for 45% of retrieved fish. Injury/trauma remained the most recorded causal category, with 46%–59% of the necropsied fish assigned to this category (see Table 3). The remaining fish were recorded with unknown and infectious disease as causal categories. Approximately 60% of the mortalities were recorded as very likely (Grade 1), and 20% each as likely (Grade 2) and most likely among the differential diagnoses (Grade 3).

External visible defects were observed in 71% (77 fish) during Visit III, and 72% (56 fish) of these fish had wounds. High degrees of scale loss (Grade 2 or 3) were seen in 63% of the necropsied fish.

#### 3.1.5 Mortality Codes Recorded

The necropsies identified 11 specific mortality causes: four from infectious diseases (category A), five from injury/trauma (category C), and two causes from the unknown category (category F). Injury/trauma was the predominant causal category, accounting for 79% of the recordings (see Table 3).

Mortality codes recorded in injury/trauma were ‘Hydrolicer/SkaMik’ (C 03.110 and C 03.140)—57% of category C, ‘handling injury unspecified’ (C 02.100)—32% of category C, ‘injury unspecified’ (C 00.100)—10% of category C and ‘operculum injury’ (C 02.170)—1% of category C. For injury/trauma, 44% of the registrations had likelihood as very likely (Grade 1), 34% as likely (Grade 2) and 21% as the most likely among the differential diagnoses (Grade 3).

Four codes were used from the causal category infectious diseases: ‘HSMI’ (A 01.160)—48% of category A, ‘viral disease not specified’ (A 01.100)—41% of category A, ‘snout and head ulcers—*Tenacibaculum* spp.’ (A 02.150)—9% of category A and ‘bacterial disease not specified’ (A 02.100)—2% of category A. Unspecified codes were recorded in Population 1 and 2, where infectious diseases had not been confirmed with diagnostics (PCR or histology). For infectious diseases, 2% of the mortalities were assigned likelihood Grade 1, 17% to Grade 2 and 81% to Grade 3.

The unknown causal category included two codes, ‘unknown’ (F 00.100)—69% of category F and ‘stunts with unknown cause’ (F 01.110)—31% of category F. For category F, 69% of the mortalities were assigned likelihood Grade 1, 12% to Grade 2 and 19% to Grade 3.

### 3.2 Mortality Causes Recorded by Farmers

**FIGURE 3:**
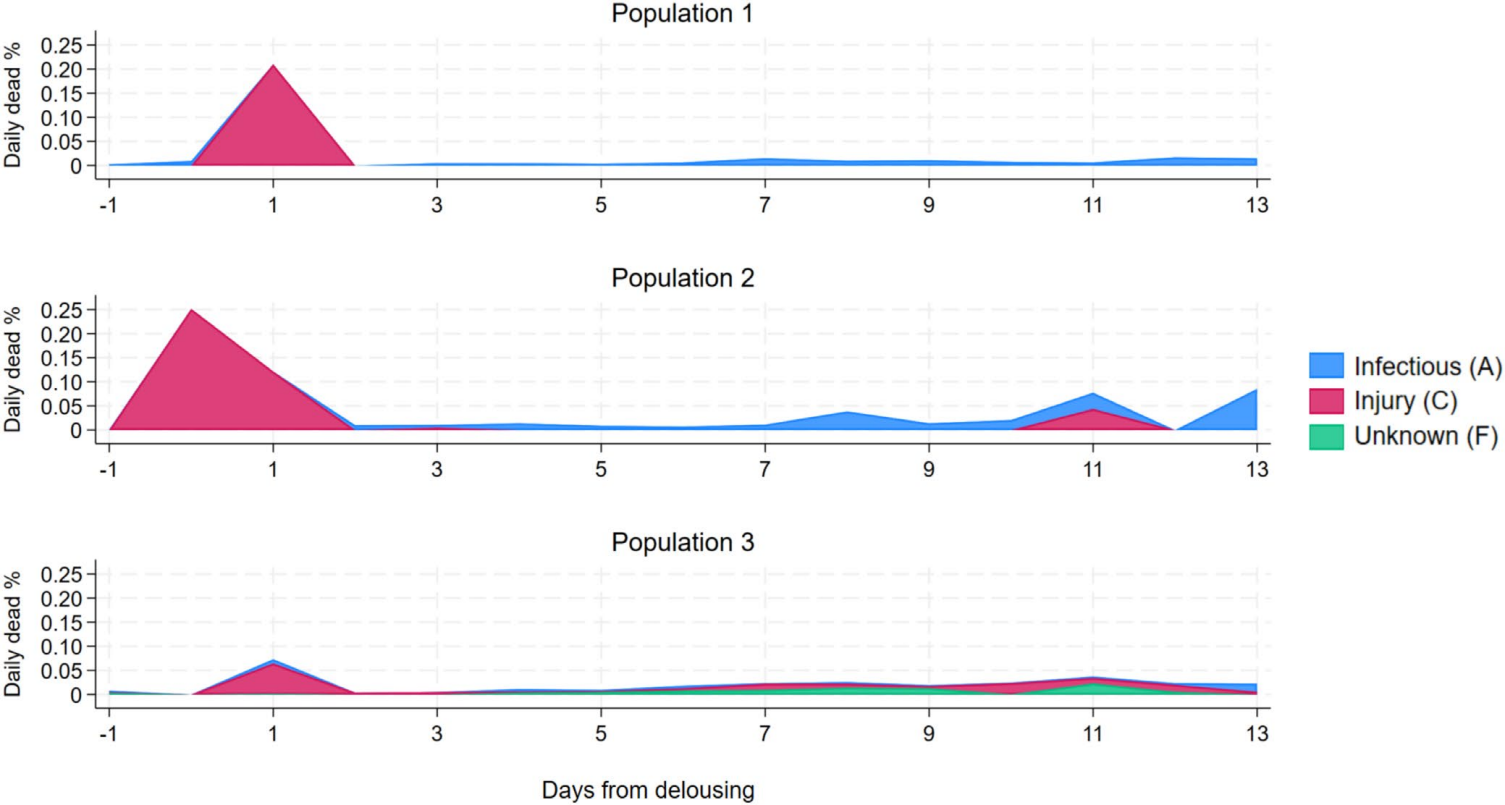
Stacked area graph illustrating the daily mortality (%) recorded by farm personnel over the 13 days following the delousing procedure. The graph shows the cause‐specific distribution of the three causal categories (A—infectious, C—injury and F—unknown) of the total mortality for each fish population. Figure created with STATA 18 and BioRender.com.

Figure 3 shows the daily mortality (%) with cause‐specific mortality fractions as recorded in the production database by the farmers from 1 day before to 13 days after the delousing. In all populations, there was an increase in recorded mortality directly after the delousing procedure. The highest mortality on the day of the delousing was seen in Population 2, with 0.25% (450 fish) and 0.12% (217 fish) mortality the following day. The lowest mortality on the day of the delousing was seen in Population 3, with 0.07% mortality (127 fish). Almost all mortality after the delousing was recorded with the delousing method as the cause of death between 1 and 3 days in the populations. Within 3 days, the daily mortality (%) decreased substantially in all populations but began to increase slightly after 6–8 days.

**FIGURE 4:**
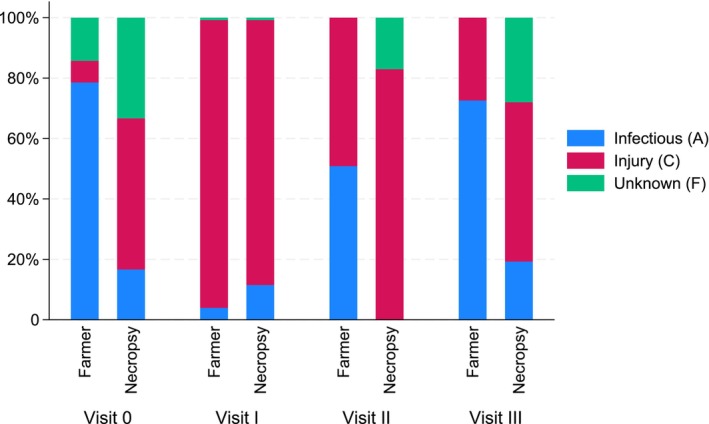
Comparison of percentages assigned to the causal categories (A, C and F) between the production database (farmer) and necropsy records (necropsy) across the four study visits (Visit 0–III). The figure illustrates the distribution (%) of fish in each category: A—infectious disease in blue, C—injury in red and F—unknown in green. Figure created with STATA 18.

Figure 4 compares percentages of fish allocated to the causal categories by farmers and in the necropsies for the days when necropsy data were available. The figure shows varying degrees of agreement between the farmers' recordings and the necropsy findings for the same days. The most significant difference between the farmer's records and the necropsy data was the duration of category C records. Compared to the necropsy data, the farmers seemed to record infectious disease (category A) causes more frequently than what was observed during necropsies. The results also showed differences between fish populations, with Population 3 having the most similar recordings to the necropsy findings. This suggests that the quality of mortality records can vary between sites and staff.

## 4 Discussion

The results showed that injury/trauma (category C) was the dominant cause of death, especially for fish that succumbed during or within 1 day after the delousing. For peracute mortality, the attributed proportion to injury/trauma in necropsied fish varied from 72% to 100%. Traumatic injuries and cerebral haemorrhages, evident from macroscopic examination, were found to be important contributors to the peracute mortalities observed. In all sites, injury/trauma remained the most frequently recorded cause of death through Visit III, suggesting that the negative effects of handling and delousing persist over time. The predominance of injury/trauma as causal category indicates that aspects of the procedures themself are largely responsible for mortalities following mechanical delousing, a finding also reported after thermal procedures (Lund, Grip, and Pettersen 2022). Comparing the cause‐specific mortality recorded by farmers to the necropsy findings shows that the farmers consistently assigned injury/trauma to a smaller proportion of fish and for a shorter time after delousing than necropsies indicated. This leads to an underestimation of causes associated with delousing and, consequently, an overestimation of other mortality causes. To accurately capture the impact of delousing events, future mortality recordings should account for the procedure's long‐term negative effects.

In this study, farmers recorded infectious diseases (category A) more frequently than necropsy results indicated. Both the count and per cent of fish categorised with infectious disease as mortality cause increased from Visit 0 to Visit 1. This could suggest that stressed or sick fish with an underlying infectious disease are less tolerant to the delousing procedure. Stress is known to affect disease susceptibility in salmon (Fast et al. 2008), but it is also discussed to be a trigger for mortality in fish with compromised health prior to delousing (Overton et al. 2018; Sviland Walde et al. 2021). A study by Vatne et al. observed that the onset of HSMI mortality coincided with a non‐medicinal delousing (Vatne et al. 2024). This study did not include diagnostic samples due to practical and economic considerations. Diagnostic results (e.g., PCR and histology) would have been valuable tools to determine the underlying cause of mortality with greater certainty. Misclassification, especially for fish classified with infectious diseases, can therefore not be ruled out. The likelihood grading in the study reflects some of this uncertainty, where 81% of the infectious disease mortalities were assigned with the most uncertain likelihood grade. Due to the low count of fish assigned to infectious diseases in this study, assessing mortality trends related to infectious diseases and delousing is challenging.

Our study observed a shift from few (in Visit I) to multiple pathological findings per fish following delousing. The few pathological changes seen during Visit I align with acute causes of death, while the multiple pathological changes can suggest a more complex pathogenesis with several pathological findings. Macroscopic findings of petechiae on gills, cerebral haemorrhages and wounds align with what has been described in other NMM studies (Gismervik et al. 2019; Thompson et al. 2023; Østevik et al. 2022). Although discoloured liver was the most occurring finding, its high prevalence could result from post‐mortem artefacts and fat accumulation. In this study, scale loss was graded higher than previously described for these delousing methods (Erikson et al. 2019; Westgård et al. 2021). However, these findings should be interpreted cautiously as the evaluation was performed on dead fish.

This study identified cerebral haemorrhages in salmon following delousing, with the majority of these lesions observed at Visit I. While it is likely that the lesions resulted from physical trauma during the procedure, other contributing factors cannot be ruled out. The exact causes of cerebral haemorrhage in salmon remain unclear, but the lesions have been reported after thermal delousing procedures (Gismervik et al. 2019; Lund, Grip, and Pettersen 2022; Poppe et al. 2018). The high incidence of cerebral haemorrhages in this study supports the theory that they are caused by injuries sustained during the procedure, possibly due to panic reactions or inadequate equipment/pipeline design.

Sedatives have been discussed as a measure to reduce mortality and improve animal welfare during delousing. Administering sedatives in commercial salmon farming can be technically difficult and time‐consuming (Folkedal, Utskot, and Nilsson 2021) and is rarely utilised due to practical considerations. Using sedatives during thermal delousing procedures has shown improved welfare and a reduction in injuries (Folkedal, Utskot, and Nilsson 2021; Thompson et al. 2023), but less is described about its effect during mechanical delousing. A study published in a Norwegian aquaculture magazine claims a potential 30% reduction in mortality with the correct use of sedatives during mechanical delousing (Costantino, Chen, and Høgset 2024). If stress or panic reactions during delousing cause the haemorrhages, sedatives may effectively reduce the occurrence and mortality from these bleedings.

Overall, the likelihood gradings in this study indicated greater uncertainty than the findings in Aunsmo et al.'s study in 2008 (Aunsmo et al. 2008). While Aunsmo et al. investigated mortality causes throughout the sea phase, this study focused on mortality related to a single incident with high mortality. Throughout a production cycle, different mortality causes are presumed to have varying degrees of certainty associated. Some causes are easily identified, while others need more extensive investigation. Some causes are also assumed to have varying degrees of certainty associated at different times in the production cycle. Likelihood grading for high‐mortality incidents, as well as throughout the production cycle, should be used to understand and improve the quality of mortality records.

Previously, the Norwegian Fish Health Reports relied solely on FHP questionnaires for information on the most common causes of mortality (Sommerset et al. 2024). Including mortality causes from the AquaCloud database in the reports enhances the accuracy of causes contributing to salmon mortality. The Norwegian Fish Health Reports are important for policymakers in the development and implementation of industry regulations. Ensuring the accuracy of the recorded mortality causes is important to guide effective resource allocation, benefiting both farmers and government authorities.

In both humans and animals, death frequently results from the interaction of multiple causes and factors. This complexity is also evident in this study, for example, when the pre‐stressed or sick fish were subjected to a stressful event like mechanical delousing. Both the infectious disease and the delousing can be seen as the underlying cause of mortality. Assigning a single mortality cause in these fish can mask the complexity and overlook essential contributing factors to mortality. In human medicine, single underlying cause registers have been criticised as an oversimplification of reality that underestimates other prevalent causes of mortality (Bishop et al. 2023). To address this, registers including multiple mortality causes, weighted according to their estimated contribution to death, have been proposed instead (Bishop et al. 2023; Piffaretti et al. 2016). Identifying the underlying cause of death is still seen as a crucial factor for understanding possibilities for prevention. Similar to the purpose of verbal autopsies in humans, salmon mortality registers aim to understand population‐level trends. Verbal autopsy is considered an important public health tool for reasonably estimating mortality causes at the populational or community level, but not necessarily at the individual level (World Health Organization 2007). Mortality causes are mainly determined at a populational level in salmon farming, and this allows for the identification of multiple causes (and contributing factors) within a population at the same time. Principles from verbal autopsies should be further explored as a model for recording salmon mortality to improve the quality of the recorded causes.

This study is the first to utilise the standardised code list of mortality and losses in aquaculture in research. The standardised code list of mortality causes has the potential to be widely used as a tool to investigate spatio‐temporal patterns of mortality. Although this study is a case series with a low amount of study units, the study demonstrates how the standardised code list can provide insight into the drivers of mortality. Based on the dominance of injury and traumatic causes seen in this study, the potential of sedation to reduce peracute mortalities after NMM delousing should be explored. Knowledge of why and when mortalities occur enables farmers to implement optimal measures at the right time to reduce mortality.

## Author Contributions

**Nanna K. Ringstad:** conceptualization, methodology, investigation, writing – original draft, visualization, data curation. **Marit Stormoen:** conceptualization, methodology, writing – review and editing, project administration, funding acquisition. **Paul J. Midtlyng:** conceptualization, methodology, writing – review and editing, funding acquisition. **David Persson:** conceptualization, methodology, writing – review and editing, funding acquisition, supervision.

## Ethics Statement

This study did not involve handling of live animals, experimental manipulations or invasive procedures.

## Conflicts of Interest

The authors declare no conflicts of interest.

## Data Availability

The data that support the findings of this study are available from the corresponding author upon reasonable request.
